# Safety and feasibility of laparoscopic gastrectomy for remnant gastric cancer compared with open gastrectomy: Single-center experience

**DOI:** 10.1097/MD.0000000000023932

**Published:** 2021-01-29

**Authors:** Junya Kitadani, Toshiyasu Ojima, Masaki Nakamura, Keiji Hayata, Masahiro Katsuda, Akihiro Takeuchi, Shinta Tominaga, Naoki Fukuda, Hideki Motobayashi, Tomoki Nakai, Hiroki Yamaue

**Affiliations:** Second Department of Surgery, Wakayama Medical University, School of Medicine, Wakayama, Japan.

**Keywords:** laparoscopic gastrectomy, open gastrectomy, remnant gastric cancer, total gastrectomy

## Abstract

The usefulness, safety and oncological validity of laparoscopic gastrectomy (LG) for remnant gastric cancer (RGC) have not been widely reported.

A total of 38 patients who underwent gastrectomy for RGC were enrolled at Wakayama Medical University Hospital between April 2008 and December 2018. All consecutive patients were included in this retrospective study; the patients were divided into the open gastrectomy group and the laparoscopic group according to the sequential nature of their operation. Fifteen patients underwent open gastrectomy for RGC (OGR) between April 2008 and December 2013, and 23 patients underwent LG for RGC (LGR) after 2014.

In the OGR group, all initial operations were performed by open surgery, whereas in the LGR group, 11 patients (47%) initially underwent laparoscopic surgery and 12 patients (53%) initially underwent open surgery (*P* = .002), 3 patients of which (25%) converted to open gastrectomy. There was no significant difference in the number of lymph node dissections or in operative time between the 2 groups, but blood loss was significantly lower in the LGR group than that in the OGR group (*P* = .002). Furthermore, although there was no difference between the 2 groups in C-reactive protein value on postoperative day 1, C-reactive protein value on postoperative day 3 was significantly lower in the LGR group than in the OGR group (*P* = .012). There were no differences in postoperative complications or long-term outcomes, including recurrence-free survival and overall survival.

LGy is suitable in cases in which the initial surgery is performed by laparoscopic surgery. Even if the initial surgery is open surgery, it is oncologically equivalent to open gastrectomy and can be performed safely with less blood loss.

## 1 Introduction

Gastric cancer is an important health problem, which is the fourth most common cancer and the second leading cause of cancer death worldwide.^[1]^ Remnant gastric cancer (RGC), which develops at the remnant stomach after gastrectomy for benign or malignant disease, is relatively rare.^[2]^ It is often diagnosed as advanced gastric cancer that is revealed after completion of standard surveillance for the initial disease. We previously reported that a periodic endoscopic follow up after prior gastrectomy was an important factor affecting the curative resection of RGC.^[3]^ Meanwhile, laparoscopic gastrectomy (LG) has become widespread, with advances in endoscopic surgical instruments and understanding of microanatomy.^[4,5]^ LG for early gastric cancer has been proven to be non-inferior in terms of safety and long-term prognosis compared with open gastrectomy (OG).^[6–8]^ Moreover, among patients with locally advanced gastric cancer, laparoscopic distal gastrectomy did have an inferior rate of disease-free survival at 3 years compared with open distal gastrectomy in a randomized clinical trial.^[9]^

LG for RGC (LGR) was first reported in 2005.^[10]^ Since then, it has been reported in several reports, but they have been limited to short-term results, and without focus on the oncological feasibility and long-term results.^[11–16]^ This retrospective study therefore compares short-term clinical outcomes regarding safety and effectiveness, and the long-term outcomes of LGR with those of open gastrectomy for RGC (OGR) in our institute.

## 2 Materials and methods

### 2.1 Patients

This retrospective cohort study was conducted at the Wakayama Medical University Hospital, Wakayama, Japan. This study was in agreement with the guidelines of the institutional ethics committee and was conducted in accordance with the Declaration of Helsinki. A total of 38 patients underwent gastrectomy for RGC at Wakayama Medical University Hospital between April 2008 and December 2018. All consecutive patients were included in this retrospective study; the patients were divided into 2 groups according to the sequential nature of their operation. Fifteen patients underwent OGR between 2008 and 2013, and 23 patients underwent LGR between 2014 and 2018. We began a prospective cohort study of LG for advanced gastric cancer in 2012 (UMIN000025029). In both the OGR group and the LGR group, clinicopathologic factors were evaluated retrospectively based on the hospital records including age, sex, initial gastric disease, type of initial gastrectomy, approach of initial operation, interval to surgery for RGC, symptom at diagnosis, tumor location, histologic type and tumor size, and surgical factors including operation time and blood loss. The clinical and pathological stages were determined according to the TNM classification (UICC 8th edition),^[17]^ and the residual tumor status was defined using the standard R-classification (R0, no residual tumor; R1, microscopic residual tumor; and R2, macroscopic residual tumor). The severity of the postoperative complications within 30 days after operation was estimated according to Clavien-Dindo classification.^[18]^

### 2.2 Surgical procedure

In both OGR and LGR, a common indication for the surgical treatment of RGC is complete resection of the carcinoma with radical lymphadenectomy. The surgery of each group of patients were performed by the same surgical teams. In principle, total gastrectomy of the remnant stomach is intended for surgically indicated RGC, but partial gastrectomy has been permitted for the elderly or high-risk cases with tumor located at the site of the anastomosis. When the initial operation was distal gastrectomy, lymph node dissection around the celiac axis, proximal splenic artery and paracardial nodes was routinely performed, and the left gastric artery was ligated at its base if it had been left undivided. Reconstruction was performed by the Roux-en-Y method. When the initial operation was proximal gastrectomy, however, the lymph node dissection around the celiac axis, infrapyloric and suprapyloric areas were routinely performed.

### 2.3 Statistical analyses

All statistical analyses were carried out using JMP Pro 14.1 (SAS Institute Inc., Cary, NC, USA). Categorical variables were assessed using Chi square method. Continuous variables were evaluated using the Wilcoxon signed-rank test. Recurrence-free survival (RFS) was calculated from the time of operation to the first documented recurrence of disease, or until death from any cause. Overall survival (OS) was defined as the time from the gastrectomy to the date of death from any cause. Survival curves were plotted according to the Kaplan-Meier method, which were subsequently compared using log-rank test. Statistical significance was defined as *P* < .05.

## 3 Results

### 3.1 Patient characteristics

Table 1 shows the demographic and clinicopathological characteristics of the LGR group compared with those of the OGR group. The patients in the LGR group were significantly older than those in the OGR group, with a median age of 74 years in the LGR group and 66 years in the OGR group (*P* = .023). There was no difference between the 2 groups in initial gastric disease, type of initial gastrectomy, reconstruction method, or interval to the surgery for RGC from the initial operation. In the OGR group, all initial operations were performed by open surgery, whereas in the LGR group, 11 patients (47%) initially underwent laparoscopic surgery and 12 patients (53%) initially underwent open surgery (*P* = .002). There were no differences between the 2 groups in terms of the tumor location, histologic type, tumor size, pathological stage, or curability.

**Table 1:** Comparison of patient characteristics between the open gastrectomy for remnant gastric cancer and laparoscopic gastrectomy for remnant gastric cancer groups.

Categories	OGR group (n = 15)	LGR group (n = 23)	*P* value
Age, median (range), yr	66 (45–79)	74 (60–89)	.023
Sex (male/female)	14/1	19/4	.339
Initial gastric disease (cancer/benign)	10/5	17/6	.630
Type of initial gastrectomy (DG/PG/PPG)	13/2/0	19/3/1	.715
Reconstruction of initial operation (B-I/B-II/R-Y/EG/Other^∗^)	11/2/0/2/0	14/3/2/2/2	.556
Approach of initial operation (Open/Laparoscopic)	15/0	12/11	.002
Interval, median (range), yr	12 (1–33)	10 (1–56)	.642
Sympton at diagnosis (yes/no)	6/9	7/16	.543
Location (anastomosis involved/non-anastomosis)	3/12	7/16	.475
Historogy (differentiated/undifferentiated)	5/10	14/9	.097
Tumor size, median (range), mm	30 (9–70)	25 (10–85)	.216
pStage UICC8th (IA/IB/IIA/IIB/IIIA/IIIB/IIIC/IV)	3/2/4/3/1/1/0/1	11/7/1/1/1/0/0/2	.114
Curability (R0/R1/R2)	13/2/0	21/1/1	.449

B-I = Billroth-I, B-II = Billroth-II, DG = distal gastrectomy, EG = esophagogastrostomy, LGR = laparoscopic gastrectomy for remnant gastric cancer, OGR = open gastrectomy for remnant gastric cancer, PG = proximal gastrectomy, PPG = pylorus-preserving gastrectomy, R-Y = Roux-en-Y.

∗ In the LGR group, there was 1 case of gastrogastrostomy after pylorus-preserving gastrectomy and 1 case of jejunal pouch interposition after PG.

### 3.2 Surgical outcomes and postoperative complications

Surgical outcomes and postoperative complications are shown in Table 2. The rates of total and subtotal gastrectomy did not differ between the 2 groups. In the OGR group, 4 patients (26%) underwent splenectomy, but none did in the LGR group (*P* = .009). There was no difference in the number of lymph node dissections between the 2 groups, but the number of metastatic lymph nodes was higher in the OGR group than in the LGR group (*P* =.014). There was no difference in operation time between the 2 groups, but blood loss was significantly lower in the LGR group than in the OGR group, with median blood loss of 115 ml in the LGR group and 290 mL in the OGR group (*P* = .002). Furthermore, although there was no difference between the 2 groups in C-reactive protein (CRP) value on the postoperative day (POD) 1, CRP value on POD3 was significantly lower in the LGR group than in the OGR group (*P* = .012). There were no differences in postoperative complication rates or hospital stay between the 2 groups, but the start time of postoperative food intake was significantly shorter in the LGR group than in the OGR group (*P* = .002). Even if the initial surgery was limited to open surgery, there was no difference in the operation time (*P* = .786) and postoperative complication rates (*P* = .721) between the OGR and LGR groups. Additionally, the blood loss was significantly smaller in the LGR group than in the OGR group (*P* = .014). There was no mortality in the OGR group and the LGR Group.

**Table 2:** Comparison of surgical outcomes between the open gastrectomy for remnant gastric cancer and laparoscopic gastrectomy for remnant gastric cancer groups.

Categories	OGR group (n = 15)	LGR group (n = 23)	*P* value
Total gastrectomy/subtotal gastrectomy	14/1	20/3	.531
Splenectomy (yes/no)	4/11	0/23	.009
Number of metastatic lymph nodes, median (range)	1 (0–8)	0 (0–6)	.014
Number of lymph node dissections, median (range)	12 (3–36)	8 (0–27)	.187
Operation time, median (range), min	281 (165–352)	302 (189–459)	.229
Blood loss, median (range), mL	290 (70–1200)	115 (15–560)	.002
Postoperative complication (all grade) (%)	6 (40)	5 (21)	.225
Postoperative complication (CD≥III) (%)	3 (20)	1 (4)	.124
Type of complication			.147
Ileus	1 (6.6)	1 (4.3)	
Pancreatic fistula	2 (13.3)	0 (0)	
Surgical site infection	2 (13.3)	0 (0)	
Roux-en-Y stasis	1 (6.6)	0 (0)	
Anastomotic stenosis	0 (0)	1 (4.3)	
Pneumonia	0 (0)	2 (8.7)	
Cerebral infarction	0 (0)	1 (4.3)	
CRP on the POD1, median (range), mg/dL	6.7 (4.1–12.1)	6.3 (3.3–11.5)	.356
CRP on the POD3, median (range), mg/dL	10.5 (3.4–20.6)	4.6 (0.9 -18.2)	.012
Postoperative food intake, median (range), d	5 (4–14)	4 (4–14)	.002
Postoperative hospital stay, median (range), d	14 (8–61)	11 (10–35)	.243

CD = Clavien-Dindo classification, CRP = C-reactive protein, POD = postoperative days.

### 3.3 Clinicopathological features of the open converted cases

In the LGR group, 12 patients (53%) underwent initial operation by open surgery, 3 (25%) of which converted to open gastrectomy due to intra-abdominal adhesions (Table 3). All 11 patients (47%) who had laparoscopic surgery from the start underwent entirely laparoscopic surgery. The initial surgery was open distal gastrectomy for a benign disease in 1case and malignant disease in 2 cases. Billroth I reconstruction was performed in 2 cases and Billroth II reconstruction in 1 case. All cases had no complications, and were discharged within 11 days after surgery.

**Table 3:** Clinicopathological features of the open converted cases.

Age	Sex	Type of previous gastrectomy	Type of previous approach	Reason for previous gastrectomy	Type of previous reconstruction	Operation time (min)	Blood loss (mL)	Tumor size (mm)	pStage (UICC8th)	Postoperative hospital stay (d)	Complication
86	F	DG	Open	GU	B-II	195	80	25	IA	11	None
68	M	DG	Open	GC	B-I	210	130	30	IIA	11	None
78	M	DG	Open	GC	B-I	287	560	20	IA	11	None

GC = gastric cancer, GU = gastric ulcer.

### 3.4 RFS and OS

**Figure 1:**
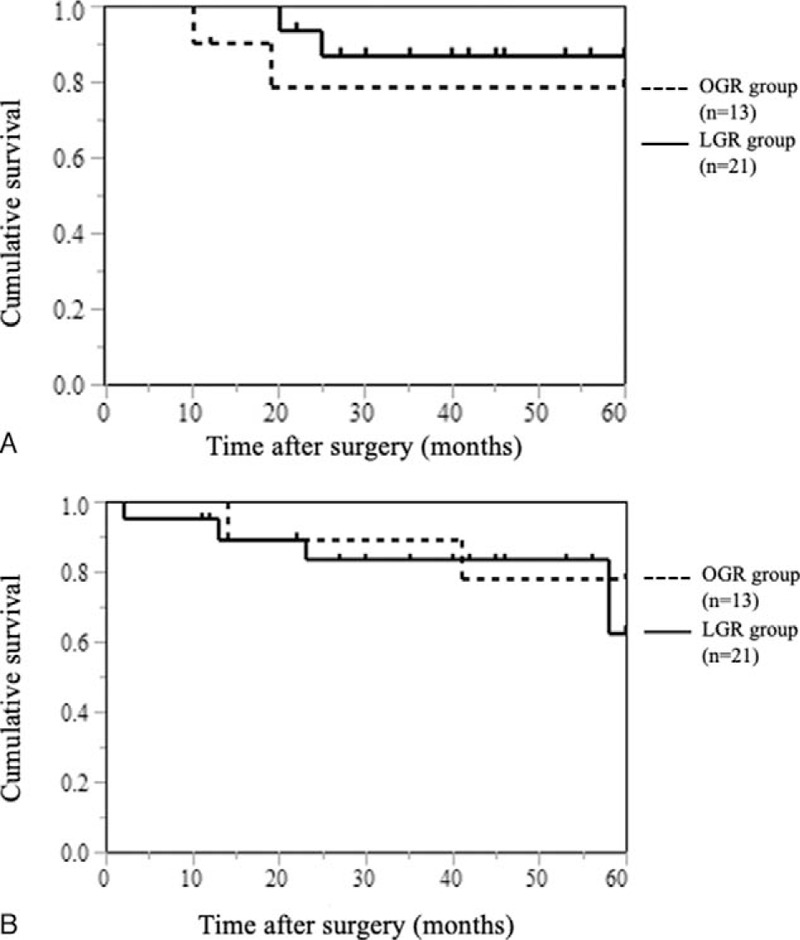
Recurrence-free survival and overall survival curves according to operative procedure. (A) Kaplan–Meier estimates of recurrence-free survival for the open gastrectomy for remnant gastric cancer (OGR) and the laparoscopic gastrectomy for remnant gastric cancer (LGR) groups. With a median follow-up period of 44 months, the 5-year recurrence-free survival rate was not different between 78% in the OGR group and 87% in the LGR group (*P* = .466 by log-lank test). (B) Kaplan–Meier estimates of overall survival for the OGR and the LGR groups. With a median follow-up period of 46 months, the 5-year overall survival rate was also not different between 77% in OGR group and 62% in LGR group (*P* = .654 by log-lank test).

We examined the RFS and the OS period of patients who underwent R0 resection. In RFS, the median follow-up period was 44 months. As shown Fig. 1A, the 5-year RFS rate was not different between 78% in the OGR group and 87% in the LGR group (*P* = .466). Two cases of recurrence were observed in both the OGR and LGR groups, respectively. In the OGR group, 1 case had peritoneal and lymph node metastasis and 1 case had peritoneal metastasis, and otherwise in the LGR group, 1 case had peritoneal metastasis and 1 case had liver metastasis. In OS, the median follow-up period was 46 months. As shown in Fig. 1B, the 5-year OS rate was also not different between 77% in OGR group and 62% in LGR group (*P* = .654).

## 4 Discussion

Surgical treatment for RGC is considered to have a high degree of difficulty due to the extensive adhesion associated with the initial surgery and the fibrosis around the dissected blood vessels and tissues, especially when the initial surgery for a malignant disease.^[11–16]^ An outstanding advantage of laparoscopic surgery is that CO2 gas of the pneumoperitoneum enters the layer to be divided, making it possible to easily and accurately confirm the separative layer, even in the patients with RGC. According to our case series, laparoscopic surgery is recommended because all operations for RGC were completed laparoscopically when the initial operation was performed laparoscopically. The total conversion rate to OGR was 13%. In each of these cases, open gastrectomy was the initial surgical approach. The rate of open conversion has been reported to be 5.6 to 47.1% in the previously published some case series. ^[11,14,15]^ The median operation time was reported 362 minutes by Nagai et al and 324 minutes by Tsunoda et al, while it was reported around 200 minutes by Kim et al and Qian et al ^[12,13,15,16]^ Even if the initial surgery was limited to open surgery, because there was an advantage in the short-term results of LGR, we make LGR the first choice as our surgical criteria for RGC.

When the depth of RGC is T3 or T4 with invasion to the greater curvature, we perform splenic hilum lymph node dissection with splenectomy. However, in the elderly and in patients with poor performance status, the spleen was preserved except when lymph node metastasis was obvious in prior imaging examination.^[19,20]^ Although the number of metastatic lymph nodes was higher in the OGR group than in the LGR group, there was no difference in the number of lymph node dissections between the 2 groups. The frequency of postoperative complications between the 2 groups was not significantly different between the groups, and LGR could be safely performed. The postoperative complication rate and the length of postoperative hospital stay was similar to the previous report.^[11–16]^ The CRP value on POD3 was significantly lower in the LGR group than in the OGR group, indicating that LGR was minimally invasive. The reason why the amount of blood loss was significantly smaller in the LGR group was considered to be that it was possible to identify blood vessels and layers to be dissected by magnified view of laparoscopic surgery.^[21]^ The prognosis of advanced RGC has been reported to be worse than that of advanced primary gastric cancer.^[22]^ No difference was found in long-term outcomes in the RFS and the OS between the 2 groups, suggesting that LGR is an oncologically appropriate surgical procedure. This study showed for the first time that laparoscopic surgery for RGC has equivalent long-term oncological outcomes compared with open surgery.

Several limitations associated with this retrospective study warrant mention. The first issue is the different historical background, that OGR was performed at the beginning of the research period, whereas LGR was performed after 2014. In this study, the patients were divided into 2 groups according to sequential nature of the operation. This design could introduce bias in the statistical analysis and reduce the power of the study. In the LGR group, median age was significantly higher than in the OGR group, and in the OGR group, the approach for the initial surgery was biased to open surgery. Secondly, the number of patients included in this study was relatively small, even though the incidence of RGC was very low. Multi-center large cohort studies evaluating short- and long-term outcomes in patients with RGC treated with LGR are required. In addition, a prospective study could overcome the limitations of the retrospective design and selection bias. Future multicenter randomized clinical trials should be designed and performed to compare the surgical approaches.

In conclusion, LGR is suitable in cases in which the initial surgery is performed by laparoscopic surgery. Even if the initial surgery is open surgery, LGR is oncologically equivalent to OGR and can be performed safely with less blood loss.

## Acknowledgments

We thank proofreading and editing by Benjamin Phillis from the Clinical Study Support Center at Wakayama Medical University.

## Author contributions

Study concept and design: Junya Kitadani, Toshiyasu Ojima.

Acquisition of data: Junya Kitadani, Masaki Nakamura, Keiji Hayata, Naoki Fukuda. Analysis and interpretation of data: Junya Kitadani, Toshiyasu Ojima, Masaki

Nakamura, Masahiro Katsuda. Drafting of the manuscript: Junya Kitadani, Toshiyasu Ojima, Masaki Nakamura, Masahiro Katsuda. Critical revision of the manuscript for important intellectual content: Hiroki Yamaue.

Statistical analysis: Junya Kitadani, Toshiyasu Ojima, Keiji Hayata, Hiroki Yamaue. Administrative, technical, and material support: Masaki Nakamura, Keiji Hayata, Masahiro Katsuda, Hiroki Yamaue. Conceptualization: Junya Kitadani, Toshiyasu Ojima.

Data curation: Junya Kitadani, Toshiyasu Ojima, Akihiro Takeuchi, Shinta Tominaga, Hideki Motobayashi, Tomoki Nakai. Formal analysis: Junya Kitadani, Toshiyasu Ojima, Masaki Nakamura. Investigation: Junya Kitadani, Toshiyasu Ojima, Masaki Nakamura, Keiji Hayata, Masahiro Katsuda.

Methodology: Masaki Nakamura. Project administration: Masahiro Katsuda, Hiroki Yamaue.

Supervision: Hiroki Yamaue. Visualization: Junya Kitadani. Writing – Original Draft: Junya Kitadani. Writing – Review & Editing: Toshiyasu Ojima.

**Conceptualization:** Junya Kitadani, Toshiyasu Ojima, Hiroki Yamaue.

**Data curation:** Junya Kitadani, Keiji Hayata, Masahiro Katsuda, Akihiro Takeuchi, Shinta Tominaga, Naoki Fukuda, Hideki Motobayashi, Tomoki Nakai.

**Formal analysis:** Junya Kitadani, Toshiyasu Ojima.

**Methodology:** Junya Kitadani, Toshiyasu Ojima, Masaki Nakamura, Keiji Hayata, Naoki Fukuda.

**Project administration:** Junya Kitadani, Toshiyasu Ojima, Hiroki Yamaue.

**Supervision:** Toshiyasu Ojima, Masaki Nakamura, Hiroki Yamaue.

**Visualization:** Masahiro Katsuda.

**Writing – original draft:** Junya Kitadani.

**Writing – review & editing:** Toshiyasu Ojima, Hiroki Yamaue.

## Abbreviations

Abbreviations: CRP = C-reactive protein, LG = laparoscopic gastrectomy, LGR = laparoscopic gastrectomy for remnant gastric cancer, OGR = open gastrectomy for remnant gastric cancer, OS = overall survival, POD = postoperative day, RFS = recurrence-free survival, RGC = remnant gastric cancer.
